# Degradation of Triazine-2-^14^C Metsulfuron–Methyl in Soil from an Oil Palm Plantation

**DOI:** 10.1371/journal.pone.0138170

**Published:** 2015-10-05

**Authors:** Ismail B. S., Eng O. K., Tayeb M. A.

**Affiliations:** School of Environmental and Natural Resource Sciences, Faculty of Science and Technology, Universiti Kebangsaan Malaysia, 43600 UKM, Bangi, Selangor, Malaysia; Institute of Zoology, Chinese Academy of Sciences, CHINA

## Abstract

Triazine-2-^14^C metsulfuron–methyl is a selective, systemic sulfonylurea herbicide. Degradation studies in soils are essential for the evaluation of the persistence of pesticides and their breakdown products. The purpose of the present study was to investigate the degradation of triazine-2-^14^C metsulfuron–methyl in soil under laboratory conditions. A High Performance Liquid Chromatograph (HPLC) equipped with an UV detector and an on-line radio-chemical detector, plus a Supelco Discovery column (250 x 4.6 mm, 5 μm), and PRP–1 column (305 x 7.0 mm, 10 μm) was used for the HPLC analysis. The radioactivity was determined by a Liquid Scintillation Counter (LSC) in scintillation fluid. The soil used was both sterilized and non-sterilized in order to observe the involvement of soil microbes. The estimated DT_50_ and DT_90_ values of metsulfuron-methyl in a non-sterile system were observed to be 13 and 44 days, whereas in sterilized soil, the DT_50_ and DT_90_ were 31 and 70 days, respectively. The principal degradation product after 60 days was CO_2_. The higher cumulative amount of ^14^CO_2_ in ^14^C- triazine in the non-sterilized soil compared to that in the sterile system suggests that biological degradation by soil micro-organisms significantly contributes to the dissipation of the compound. The major routes of degradation were O-demethylation, sulfonylurea bridge cleavage and the triazine “ring-opened.”

## Introduction

The environmental fate of pesticides in the soil is viewed with great concern today, due to the potential effects on surface and ground water quality. The rate of mobility and degradation in the soil are the most important processes that determine the fate of pesticides in soils. Therefore, degradation studies in soils are essential for the evaluation of the persistence of pesticides and their breakdown products. Data on the rate of degradation are extremely important as they permit prediction of the levels likely to remain in soil and assessment of potential risk associated with exposure. Under favourable conditions, microbial degradation of pesticides should be studied under field conditions but this is often dogged by problems due to the interplay of several factors of spatial and temporal variability. Laboratory degradation studies have the merit of being carried out under controlled conditions which allow for the measurement of one factor at a time. This approach may permit the detection of which factor is more responsible for the dissipation of a particular pesticide, within a given environment and soil. When properly planned, laboratory studies have been found to produce good data that can be employed in modelling pesticide degradation in the field. Sulfonylurea herbicides degrade in soils primarily by chemical hydrolysis and microbial metabolism and there are several publications, which elucidate the significance of microbial degradation [1–4]. Chemical hydrolysis of metsulfuron-methyl has been shown to be very rapid at low pH. This means it is less persistent in soils with low pH [5]. Its persistency at 45°C increased from 2.1 days at pH 5 to 33 days at pH 7 [6]. The degradation rate of metsulfuron-methyl is affected by soil temperature, moisture, pH, and soil microbial viability [7]. The half-life of metsulfuron- methyl ranges from 2.5 days [soil conditions: pH 3.1, 35°C, 80% field water holding capacity (FC)] to 36 days (soil conditions: pH 5.7, 10°C, 60% FC) depending on the above-mentioned factors [1, 8, 9]. The degradation rate of metsulfuron-methyl has been positively correlated with microbial biomass [8, 10]. Although several researchers have reported the effects of environmental conditions on the degradation rates of metsulfuron-methyl, there have been only a few attempts to identify the degradation products. Some aqueous hydrolysis products, plant metabolites, and degradation products of metsulfuron-methyl in soil minerals and humic acids have been identified [3, 11–14]. The objective of the present study is to determine the degradation rate and pattern of ^14^C-labelled metsulfuron-methyl in sterile and non-sterile Bernam Series soil under laboratory conditions. The Bernam Series soil was selected for the study, and observations from the study will be further verified in the field (with the same soil series). ^14^C-radiollabelled metsulfuron-methyl labelled at the 2-carbon in the triazine ring [triazine-2-^14^C] and phenyl ring [phenyl-^14^C] was used in the study to identify its metabolic pathway in the soil.

## Materials and Methods

### Chemicals

The herbicide selected for the study was metsulfuron-methyl. The purity of the technical sample used as the analytical standard and for various laboratory studies was approximately 99%. ^14^C-radiolabelled metsulfuron-methyl (methyl 2-[[[[(4-methoxy-6-methyl–1, 3, 5-triazin-2-yl) amino] carbonyl] amino] sulfonyl] benzoate) was synthesised at DuPont, New England Nuclear (NEN) Research products, Boston MA. It was labelled uniformly at the 2-carbon in the triazine ring [triazine-2-^14^C] and phenyl ring [phenyl-^14^C] and has specific activity of 1.85 MBq mg–1 (49.87 μCimg^−1^) and 1.42 MBq mg–1 (38.28 μCimg^−1^), respectively. The ^14^C-labelled compound used had radiochemical purity higher than 99%, as determined by high performance liquid chromatography (HPLC). Unlabelled reference standards of the test substance and expected degradation products were synthesised at the DuPont Agricultural Products, E.I. Du Pont de Nemours and Company (Wilmington, DE). All organic solvents and water used in the study were of HPLC grade. All other chemicals were of the deionized grade. The radioactivity in the samples was determined by LSC in scintillation fluid. The potassium hydroxide and ethylene glycol trap solution was used to trap ^14^CO_2_ and organic volatiles released during combustion.

### Soil

Soil samples (Bernam Series) were collected from the top 15 cm of the soil at the field trial site at Sungai Buloh Estate. Soil characteristics were determined at the Harris Laboratories, Inc. (Lincoln, NE), and are provided in Table 1. The fresh soil was sieved through a 2 mm sieve and used immediately for the study. Soil samples taken from biometer flasks identical to those used in the degradation studies were evaluated for total bacterial counts so as to ascertain the effect of the closed system of the flask on the microbial population of the soils. The soil was sampled at day 0 (immediately after treatment), and at 30 and 60 days. The plating process commenced with the measurement of 23.0 g of nutrient agar (from Difco laboratories, Detroit MI 48232–7058, USA) consisting of Bacto beef extract (3 g), Bacto peptone (5 g) and Bacto agar (15 g) to which was added to 800 mL of sterilised water. The mixture was heated on a hot plate magnetic stirrer until uniform solubility was achieved and the final solution was made up to 1 litre with sterilised water. An autoclave (All American Electric Pressure Steam Sterilizer Model No 25X) was used to sterilise the agar solution (at 15 kPa for 15 minutes, temperature 121°C). The agar solution was left to cool at room temperature soon after autoclaving, and while still warm, plating was done. Agar plating was carried out using 25 mL of agar solution per plate, left overnight to solidify. Soil samples (10 g each) collected at specified intervals of time, were added to conical flasks each containing 90 mL of sterilised water and shaken on a rotary shaker (Stuart Flask Shaker; Stuart Scientific Co. Ltd. England) for 1 h. This was followed by serial dilution of 10 fold steps and the appropriate dilutions poured out onto nutrient agar plates and incubated for a duration of 24–48 h at 30°C, before counting of the colonies was carried out. Results of microbial colonies counted were expressed in colony forming units (CFU).

**Table 1:** Soil characterization.

Soil Property	Unit	Result
Texture	NA^2^	Clay
Sand	%	27.6
Silt	%	27.2
Clay	%	45.2
pH (Water)	NA	4.5
pH (0.01 M CaCl_2_)	NA	4
Organic Matter (Ashing)	%	4.6
Organic Matter (Walkley-Black)	%	4.3
Organic Carbon (Walkley-Black)	%	2.4
Bulk Density	g/cm^3^	0.92
Moisture Holding Capacity (0 Bar) ^3^	%	65.2
Moisture Holding Capacity (0.1 Bar)	%	46.5
Moisture Holding Capacity (1/3 Bar)	%	45.0
Moisture Holding Capacity (15 Bar)	%	23.3
Cation Exchange Capacity	meq/100 g	25.6
Nitrogen, Total	mg/kg	2220.00
Phosphorus (Bray)	mg/kg	96
Phosphorus (Olsen)	mg/kg	39
Potassium	mg/kg	313
Magnesium	mg/kg	61
Calcium	mg/kg	763
Sodium	mg/kg	22
Soluble Salts	mmhocm^-^ ^1^	0.23

^1^ USDA system (Sand: 2 mm–50 μm, Silt 50–2 μm, Clay: <2 μm)

^2^ NA = Not Applicable

^3^% of dry weight

### Experimental Setup

**Fig 1:**
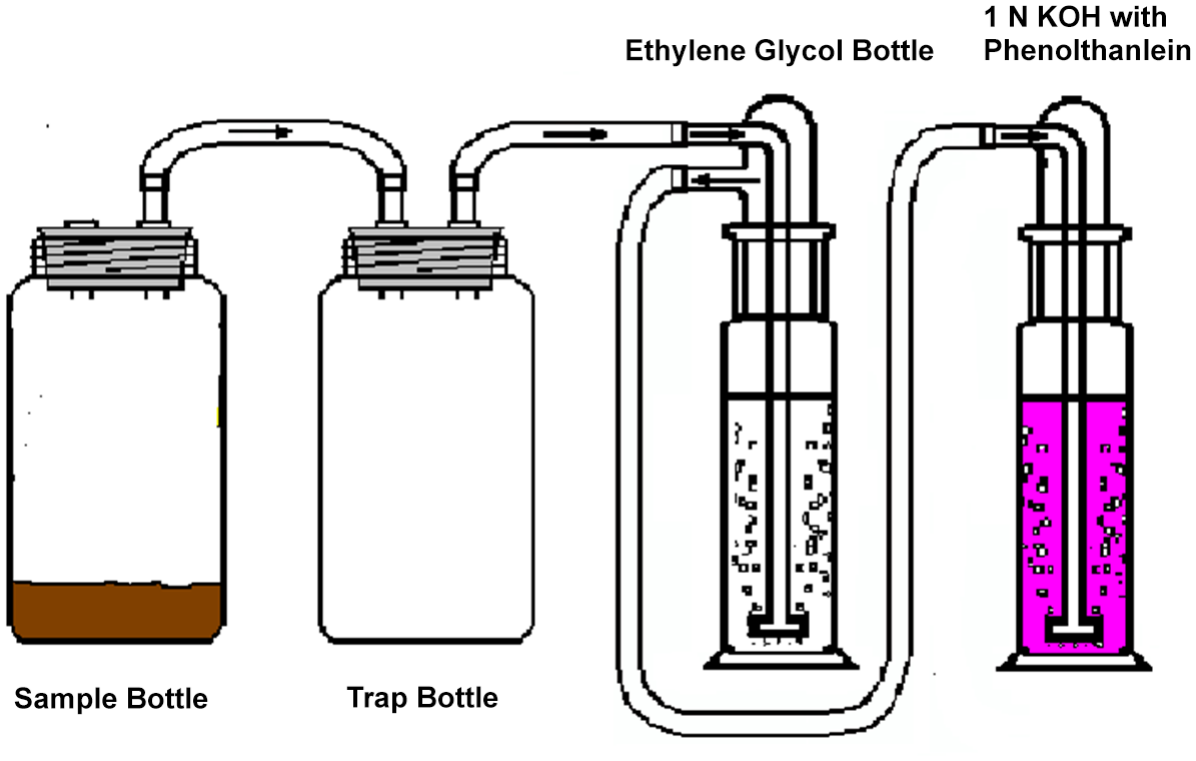
Experimental set-up for the study of metsulfuron-methyl degradation in soil.

Samples of the Bernam Series soil was collected from the field experimental site at Sungai Buloh Estate. The soils samples were passed through a 2 mm sieve and used immediately. Each soil sample (100 g) was placed in a sample bottle, connected to the trap bottle. The trap bottle was connected to an ethylene glycol (25 mL) bottle to trap any organic volatiles. Lastly the ethylene glycol bottle was connected to a 0.1 M KOH (25 mL) bottle to trap ^14^CO_2_ evolved as a result of microbial activity (Fig 1). The flask was corked with a rubber stopper. The soil was maintained at the water holding capacity of 50% throughout the experiment. This was achieved by periodically weighing the flask and by the addition of water as required. The experiment was conducted in a “controlled environment” chamber with the temperature maintained at 30°C (±1°C) and the humidity at 80% (±2%), in total darkness. The experiment was replicated three times.

For the sterile system, the same number of flasks was prepared as described above except that each flask that contained 100 g dry weight equivalent (of the moist soil) which was autoclaved at 15 kPa and 120°C for 1 hour on four separate days to destroy the microorganisms. The Market Forge Sterilmatic autoclave Model STM-EL was used to sterilise the soil. The sterile test system was connected to a volatile trapping system as described above, with the addition of a sterile Gelman ACRO 50 PTFE 0.2 μm filter inserted before the test system flask, to prevent microbial contamination.

### Treatments

#### Triazine Ring [triazine-2-14C] Metsulfuron-methyl

The primary stock solution of 1503 μg/mL (specific activity, 49.87 μCi/mg) was prepared by mixing 15.03 mg of the neat material (99% purity) with 10 mL of sterilised water where the pH was adjusted to a pH of 7. This primary stock solution was stored frozen when not in use. A 15.03 μg/mL secondary stock solution (specific activity, 49.87 μCi/mg) was prepared by diluting a 1 mL aliquot of the primary stock solution (1503 ppm) to a final volume of 100 mL in sterile deionized water. A 93 μL aliquot of this solution was mixed with 100 g of soil in each designated vessel to produce a concentration in the soil of approximately 1.4 ppm. This concentration allowed for adequate detection levels of the parent material and its metabolites.

#### Reference Standard Solutions

Solutions of metsulfuron-methyl and reference chemicals were prepared by dissolving 5 mg of the mixture in 5 mL of water: acetonitrile (3:2, v: v). Dilutions and mixtures of the standard solution were prepared in reagent water prior to the HPLC analysis. Mixtures of the following standard solutions were prepared to evaluate the HPLC resolution of degradation from the triazine radiolabelled test substance and for the purpose of performing chromatography: IN-B5528, IN-A4098, IN-D5803, IN-B5067, IN-F5438, IN-MU717, and DPX-T6373.

#### Sample Collection and Handling

Sampling was conducted at various intervals by taking the three replicate bio meter flasks at 0 (immediately after treatment), 1, 3, 7, 10, 14, 21, 30, 45, and 60 days after treatment (DAT). One mL aliquots (in triplicates) of 0.1 M KOH and ethylene glycol solutions used to trap ^14^CO_2_ and organic volatiles were sampled at each of the specified intervals. The solutions were combined with the scintillation fluid and analysed for total radioactivity by the Liquid Scintillation Counter (LSC).

#### Extraction of Soil Radioactivity

At each sampling time, two flasks (one from each of the two ^14^C metsulfuron-methyl treatments) were taken for the extraction process. Step 1—The soil in each test flask was mixed with 100 mL of acetonitrile: 2 M ammonium carbonate (9:1, v: v) and shaken for 1 hour on a platform shaker at room temperature (23°C). The solution was centrifuged at approximately 2500 rpm for 15 minutes. The supernatant was decanted to a graduated cylinder. The extraction process was conducted 3 times, and the extracts pooled; 1 mL aliquots were analysed in triplicate by LSC. The recovery study was conducted by spiking a known amount of two ^14^C metsulfuron-methyl on the soil sample following the same procedure. The sample was left for 1 hour before it was analysed by LSC.

After step 1 extractions were done, the bound residue (%) in the extracted soil was estimated from the equation:

Estimated Bound Residue (%) = 100 - % Applied Radioactivity in Traps - % Applied Radioactivity in Extracts

If the estimated bound residue was >10% of the applied radioactive metsulfuron-methyl, extraction step 2 was done. Step 2—After the first extraction, extraction of the remaining residues from the soil samples were done using 100 mL of CH_2_Cl_2_: methanol: 2 M ammonium carbonate (3:4:1, v: v: v), followed and by shaking for 1 hour at room temperature (23°C). The extraction process was conducted 3 times, the extracts pooled, and 1 mL aliquots were analysed in triplicate by LSC. The LSC aliquots must be withdraw in the homogenous phase. After step 2 was completed, the step 1 and step 2 extracts were pooled. The extracts were concentrated by vacuum rotary evaporation and re-dissolved in water. Aliquots were analysed in triplicate by LSC and an aliquot was removed and analysed by HPLC. The extracts were stored in a freezer at—4°C.

#### Analysis of Soil Extracts

Soil extracts were analysed using a HPLC equipped with both a UV—detector and an on-line radio-chemical detector (Ramona, Raytest Inc). The HPLC method 1 used the Supelco Discovery column (250 x 4.6 mm, 5 μm) and a gradient with mobile phase A, a phosphate buffer H_2_O (pH ± 7), and B, methanol (from 0 to 3.5 min, 100% B; at 19 min, 90% B; at 19.5 to 29.5 min, 80% B; at 30 to 37 min, 65% B; at 40 to 42 min; 0% B and 45 min, 100% B). The HPLC method 2 used a PRP–1 column (305 x 7.0 mm, 10 μm) with the same mobile phase as the HPLC method 1 but with a different gradient (from 0 to 3 min, 10% B; at 10 min, 20% B; at 20 min, 40% B; at 30 min, 90% B at 35 min, 100% B). The mobile phase flow rate was 1.5 mL min^−1^ for both methods. The oven and injector temperature readings were set at 35°C. The HPLC method 1 was used for all the sample analyses and HPLC method 2 was used for confirmatory analyses. The standard mix (a mixture of available standards) was used to verify that the above HPLC conditions adequately separated metsulfuron-methyl from its expected metabolites, and also to confirm the retention times of the standards used during the course of the study. Aliquots of concentrated extracts of 250 μL were injected onto the column, and the elution of radioactivity was monitored and quantitated by fraction collection and LSC.

#### Determination of Soil Un-extractable residues

Post-extracted soil samples were air-dried in the laboratory hood. When dry, the samples were homogenized and weighed. Aliquots in triplicate were combusted using the Harvey biological oxidizer (Harvey Instrument Inc., model OX 500). The ^14^CO_2_ released from the combustion process was trapped in 15 mL of ^14^C-cocktail scintillation fluid and radioactivity was measured from the LSC analysis.

#### Determination of DT50 and DT90

The first-order model was used to estimate the DT_50_ (the time required for 50% of the applied chemical to degrade) and DT_90_ (the time required for 90% of applied chemical to degrade) values. The First-order model equation used is as [15–16]:

C=CO.e−K*t

Where,

C0: Initial concentration (mg/kg)

C: Concentration at time t (mg/kg)

K: Degradation rate constant

## Results and Discussion

### Assay for Total Microbial Population

Microbial plate counts from the Bernam Series soil samples conducted at day 0, 30 and 60 are presented in Table 2. The soil microorganisms remained viable throughout the study in the non-sterile flasks. There was a slight increase of microbial mass at day 60. These observations indicated that the non-sterile system under the degradation studies that were conducted, had no adverse effect on the microbial population. An earlier study by Ismail *et al*. [17] demonstrated that application of metsulfuron-methyl would not adversely affect soil microbial biomass and population once the soil microbes adapted to the presence of metsulfuron-methyl.

**Table 2:** Bacterial counts in the non-sterile soil samples.

Incubation Time (day)	Bacterial Counts (CFU/ g soil X 10^−5^)
0	1.33 (± 0.25)
30	5.36 (± 0.07)
60	8.23 (± 0.13)

#### Radiolabelled triazine treated soil samples

**Fig 2:**
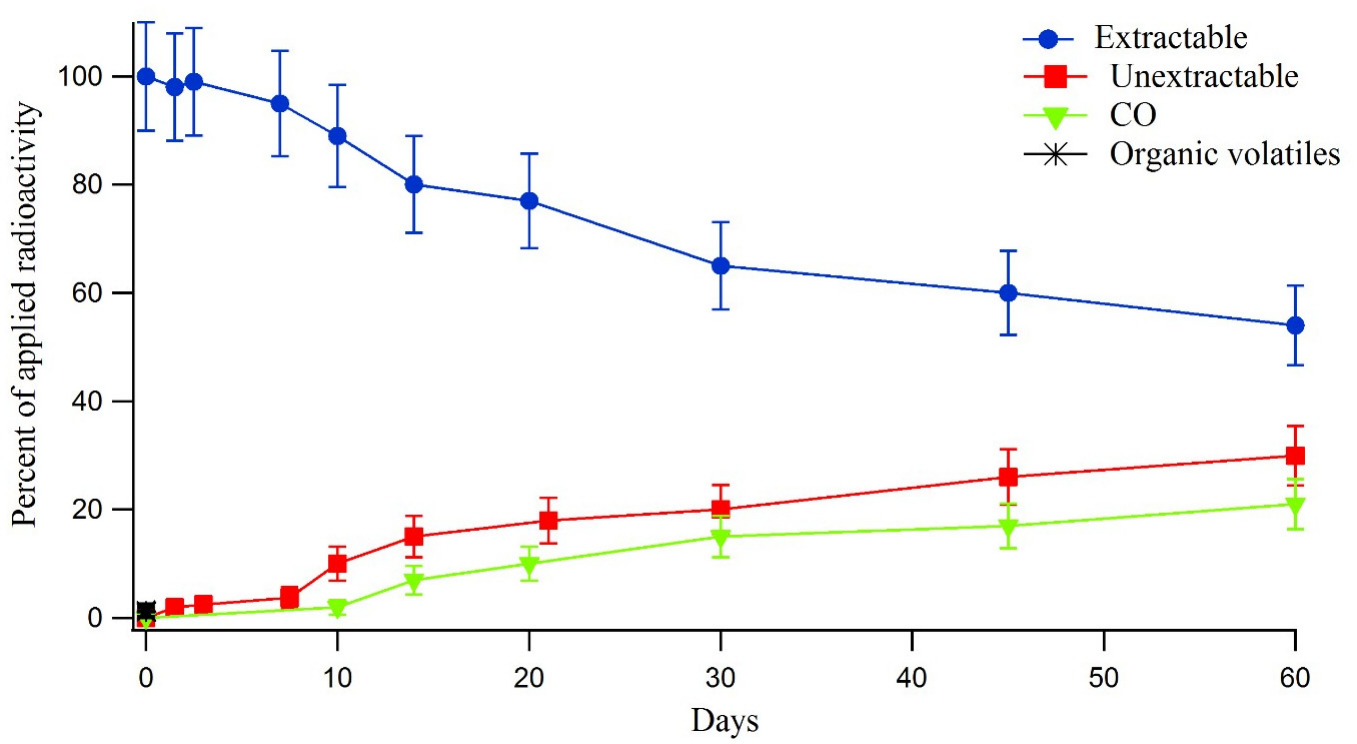
Distribution and recovery of radioactivity in the non-sterile soil system treated with ^14^C-triazine metsulfuron-methyl.

**Fig 3:**
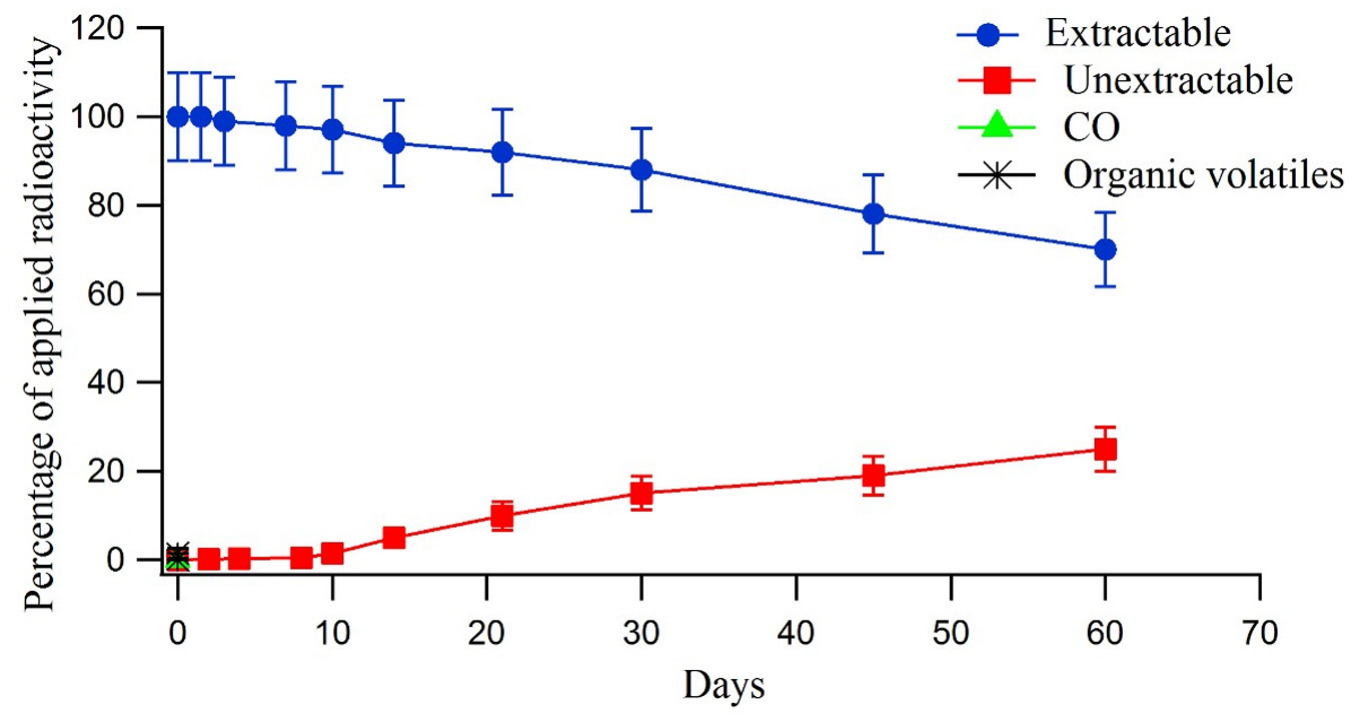
Distribution and recovery of radioactivity in the sterile soil system after treatment with^14^C-triazine metsulfuron-methyl.

Data on the recovery and distribution of radioactivity from the non-sterile and sterile soil samples treated with ^14^C-triazine metsulfuron-methyl are presented in Figs 2 and 3 respectively. The recovery (mass balance) of applied radioactivity for the ^14^C-triazine main test system soil samples ranged from 97.6% to 101.7% with an average of 100.1 ±3.3%. The total recovery of applied radioactivity (mass balance) for the ^14^C-triazine sterile test system soil samples ranged from 91.6% to 101.9% with an average of 97.8 ±5.6%. The extracted radioactivity from the non-sterile system treated with ^14^C-triazine metsulfuron-methyl steadily decreased with time from 100% to 48% at day 60. The formation of ^14^CO_2_ corresponded to the decline of the parent material; ^14^CO_2_ increased to about 24% of the applied radioactivity at day 60. Un-extractable residues that increased consistently throughout the experiment accounted for about 30% of the applied radioactivity at day 60. Less than 0.1% of the applied radioactivity was classified as organic volatiles (Fig 2).

In the sterile system treated with ^14^C-triazine metsulfuron-methyl, the extracted radioactivity steadily decreased with time from 100% to 66% at day 60. Cumulative amounts of ^14^CO_2_ were only recorded at day 60 and accounted for 0.5% of the applied radioactivity. Un-extractable residues steadily increased to about 26% of the applied radioactivity at day 60. Organic volatiles were not detected throughout the experiment (Fig 3). The same trend was seen in the ^14^C-triazine metsulfuron-methyl where in the non-sterile system, the amount extracted from applied radioactivity showed faster degradation than that in the sterile system and the ^14^CO_2_ release was also faster in the non-sterile system compared to that in the sterile system. In the non-sterile system, the rate of ^14^CO_2_ release was faster in the soil treated with ^14^C-phenyl metsulfuron-methyl (28.3% of the applied radioactivity at day 60) than that in soil treated with ^14^C-triazine metsulfuron-methyl (23.6% of the applied radioactivity at day 60). The different rates of mineralization indicate that the phenyl moiety is more susceptible to microbial attack than the triazine moiety on the molecule [18–19].

#### Rate of Degradation of Metsulfuron-methyl in the Bernam Series Soil

**Fig 4:**
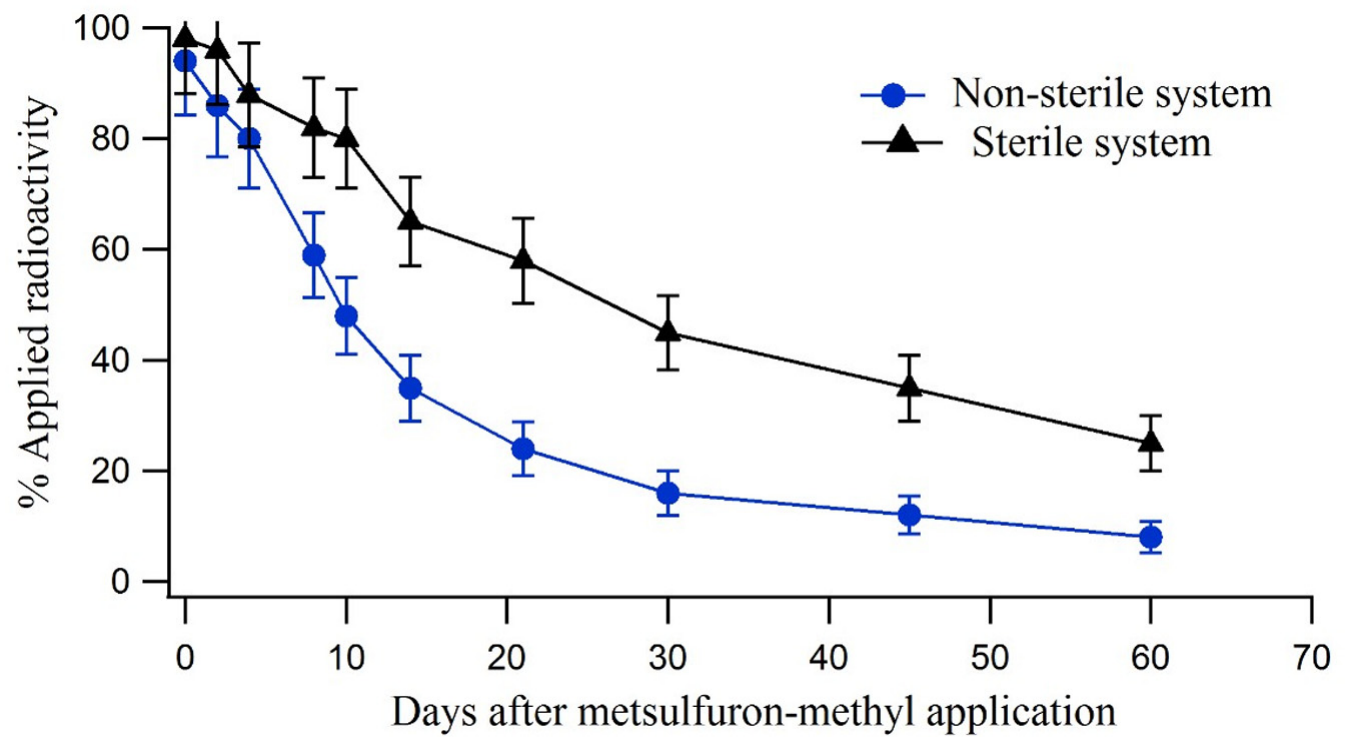
Dissipation of ^14^C triazine metsulfuron-methyl in soil over time.

The first-order model was used to determine the DT_50_ and DT_90_ values of ^14^C-triazine metsulfuron-methyl in both the non-sterile and sterile systems (Fig 4). The DT50 was estimated by using the equation:*DT*_50_ = *In* (2)/*K*. The degradation rate constant (k) was determined using linear regression of ln (*C*/*CO*) (C = concentration) over time and was the slope of the linear regression line. Based on the first-order model, the ^14^C-triazine non-sterile system had DT_50_ and DT_90_ of 14 days and 47 days respectively. The ^14^C-triazine sterile system had DT_50_ and DT_90_ of 23 and 76 days (Table 3). These results are similar to the findings of Zhang and Yin [20] where it was reported that half-lives of 8 to 36 days (based on the first-order model) were obtained in an acidic soil (pH 5.7 and 7.3% Organic Carbon) at various soil moisture and temperature levels.

**Table 3:** Degradation of ^14^C-triazine metsulfuron-methyl in Bernam Series Soil—First-order Model.

System	First-order	Model	DT_50_ (day)	DT_90_ (day)
	K (slope)	r^2^		
Non-sterile	0.0493	0.97	14	47
Sterile	0.0320	0.99	23	76

The DT_50_ values of metsulfuron-methyl in the non-sterile system were lower than those in the sterile system, suggesting that biological degradation (by soil microorganisms) significantly contributed to the dissipation of this compound. The dissipation of metsulfuron-methyl in the soil involves both chemical and microbial processes, and this has been observed in the behaviour of other sulfonylurea herbicides as well [21–23]. The degradation of metsulfuron-methyl in an acidic soil environment is relatively rapid because of sulfonylurea bridge hydrolysis which diminishes in alkaline soils.

#### Soil Degradation of Metsulfuron-methyl

The composition of the radioactivity in the soil samples treated with ^14^C-triazine metsulfuron-methyl, are presented in Tables 4 and 5 for the non-sterile and sterile systems. In the ^14^C-triazine metsulfuron-methyl non-sterile system, seven metabolites were detected. Five metabolites were identified as B5528, A4098, NC 148, B5067 and F5438. The other two metabolites, T3 and T7 could not be identified. The chemical name and structure of the identified metabolites are shown in Appendix B4. Six metabolites were detected in the sterile system, four metabolites, B5528, A4098, NC148 and B5067 were detected in the non-sterile system. Re the other two metabolites, MU717 and ST4, MU717 was the only metabolite different from the metabolites in the non-sterile system whereas ST4 could not be identified.

**Table 4:** Degradation of ^14^C-phenyl metsulfuron-methyl in the Bernam Series soil samples in the non-sterile system.

Times	Applied Radioactivity (%)															
(Day)	T6373	1.00	T1	0.10	T2	0.40	T3	0.63	T4	0.68	T5	0.73	T6	0.83	T7	0.86
			IN-B5528		IN-A4098		UN		IN-NC148		UN		IN-B5067		IN-F5438	
0	93.3	± 9.16	2.5	± 0.16	3.4	± 0.26	ND	ND	ND	ND	ND	ND	ND	ND	ND	ND
1	86.8	± 7.93	4.3	± 0.35	ND	ND	ND	ND	ND	ND	ND	ND	6.2	± 0.72	ND	ND
3	79.2	± 6.18	5.3	± 0.41	ND	ND	ND	ND	ND	ND	ND	ND	13.6	± 1.10	ND	ND
7	58.1	± 3.89	12.2	± 1.13	ND	ND	6.3	± 0.46	ND	ND	ND	ND	12.8	± 1.32	2.1	± 0.11
10	46.8	± 3.21	12.8	± 1.18	ND	ND	6.9	± 0.49	1.5	± 0.05	ND	ND	16.5	± 1.43	1.2	± 0.04
14	33.7	± 3.05	ND	ND	14.7	± 1.36	3.6	± 0.32	6.5	± 0.73	2.4	± 0.20	14.3	± 1.22	3.1	± 0.28
21	23.3	± 2.27	ND	ND	22.2	± 2.42	5.3	± 0.43	5.8	± 0.51	4.5	± 0.33	10.4	± 1.03	2.3	± 0.20
30	14.1	± 1.25	ND	ND	26.6	± 2.75	4.3	± 0.38	3.5	± 0.23	3.5	± 0.27	6.5	± 0.83	5.3	± 0.43
45	10.1	± 0.83	ND	ND	24.2	± 0.68	4.8	± 0.41	5.6	± 0.47	3.4	± 0.22	5.3	± 0.51	2.6	± 0.19
60	4.8	± 0.32	ND	ND	23.8	± 0.52	3.3	± 0.28	6.3	± 0.66	4.2	± 0.31	3.7	± 0.23	1.8	± 0.03

UN = Unknown. ND = Not detected. T6373 = 2-[3-(4-methoxy-6-methyl–1, 3, 5-triazin-2-yl-ureidosulfanoyl)-benzoate acid methyl ester (metsulfuron-methyl). B5528 = 4-amino-6-methyl–1, 3, 5-triazine-2-ol. A4098 = 4-methoxy-6-methyl1-1, 3, 5-triazine-2-amine. NC148 = Not available. B5067 = Methyl 2 [[[[(4-hydroxy-6-methyl–1, 3, 5-triazine-2y 1) amino] carbonyl] amino] sulfonyl] benzoate. F5438 = Methyl 2 [[[[(4-methoxy-6-methyl–1, 3, 5-triazine-2y 1) amino] carbonyl] amino] sulfonyl] benzoate acid.

**Table 5:** Degradation of ^14^C-phenyl metsulfuron-methyl in the Bernam Series soil samples in the sterile system.

Times	Applied Radioactivity (%)													
(Day)	T6373	1.00	ST1	0.10	ST2	0.40	ST3	0.68	ST4	0.73	ST5I	0.83	ST6I	0.96
			IN-B5528		IN-A4098		IN-NC148		UN		N-B5067		N-MU717	
0	96.1	± 8.65	1.3	± 0.02	ND	ND	ND	ND	ND	ND	2.1	± 0.15	ND	ND
1	94.3	± 8.23	1.5	± 0.03	ND	ND	ND	ND	ND	ND	3.6	± 0.23	ND	ND
3	88.9	± 8.11	3.8	± 0.28	ND	ND	ND	ND	ND	ND	6.5	± 0.43	ND	ND
7	80.2	± 7.36	8.3	± 0.73	ND	ND	ND	ND	ND	ND	7.3	± 0.57	2.1	± 0.12
10	77.9	± 6.28	6.3	± 0.58	ND	ND	ND	ND	ND	ND	12.2	± 1.33	1.2	± 0.02
14	63.0	± 5.22	5.0	± 0.46	8.3	± 0.72	1.5	± 0.08	ND	ND	13.8	± 1.40	3.1	± 0.22
21	52.7	± 4.58	3.8	± 0.22	11.9	± 1.23	2.3	± 0.12	1.5	± 0.03	17.3	± 1.54	2.3	± 0.26
30	37.8	± 3.48	1.8	± 0.08	20.5	± 1.78	3.1	± 0.23	ND	ND	13.3	± 1.22	15.2	± 1.32
45	27.0	± 2.18	2.3	± 0.13	21.9	± 1.93	2.1	± 0.10	ND	ND	12.8	± 1.18	9.4	± 85
60	15.5	± 1.66	3.1	± 0.26	24.3	± 2.15	1.5	± 0.05	ND	ND	10.1	± 1.06	11.3	± 1.02

UN = Unknown. ND = Not detected. T6373 = 2-[3-(4-methoxy-6-methyl–1, 3, 5-triazin-2-yl-ureidosulfanoyl)-benzoate acid methyl ester (metsulfuron-methyl). B5528 = 4-amino-6-methyl–1, 3, 5-triazine-2-ol. A4098 = 4-methoxy-6-methyl1-1, 3, 5-triazine-2-amine. NC148 = Not available. B5067 = Methyl 2 [[[[(4-hydroxy-6-methyl–1, 3, 5-triazine-2y 1) amino] carbonyl] amino] sulfonyl] benzoate. MU717 = Not available.

#### Radiolabelled triazine treated soil samples

**Fig 5:**
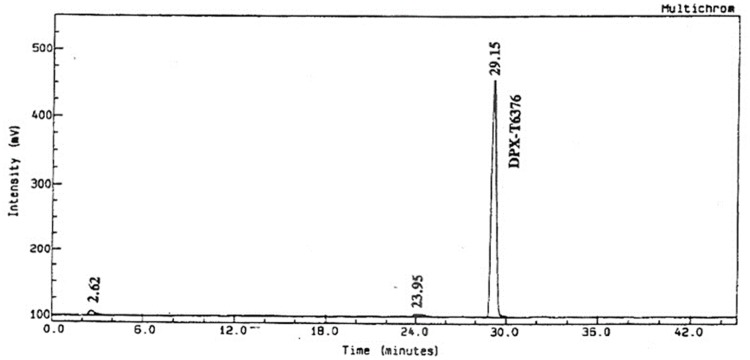
^14^C-radiochromatogram of ^14^C-triazine metsulfuron-methyl in the non-sterile system (on day 0).

**Fig 6:**
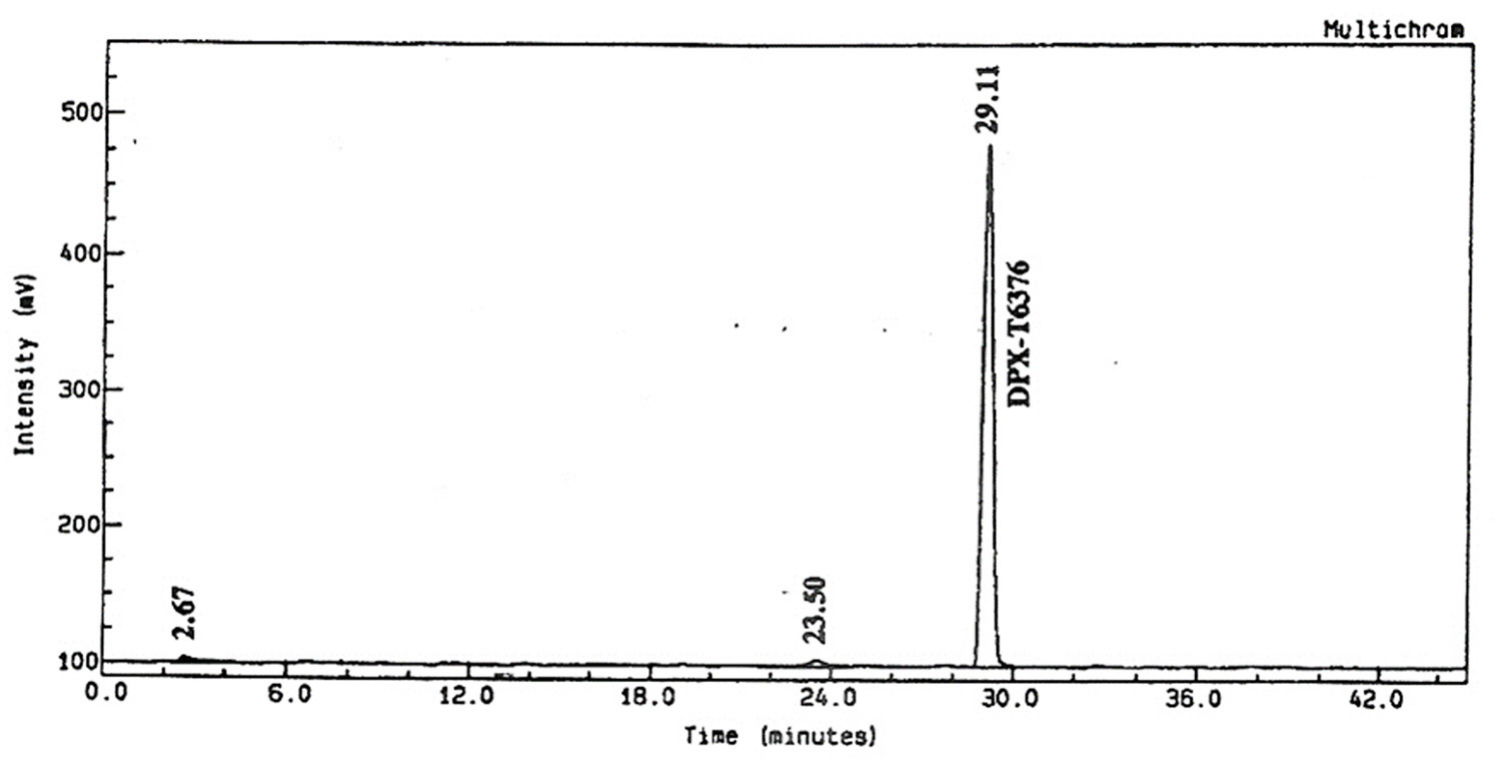
^14^C-radiochromatogram of ^14^C-triazine metsulfuron-methyl on day 0 samples in the sterile system.

The analysis on day 0 soil extracts from the fraction collector indicated that 93.3% and 96.1% of the applied radioactivity was recovered as ^14^C-triazine metsulfuron-methyl from the non-sterile and sterile systems respectively (Figs 5 and 6). These results demonstrated that the test substance was stable in the systems used for extraction.

**Fig 7:**
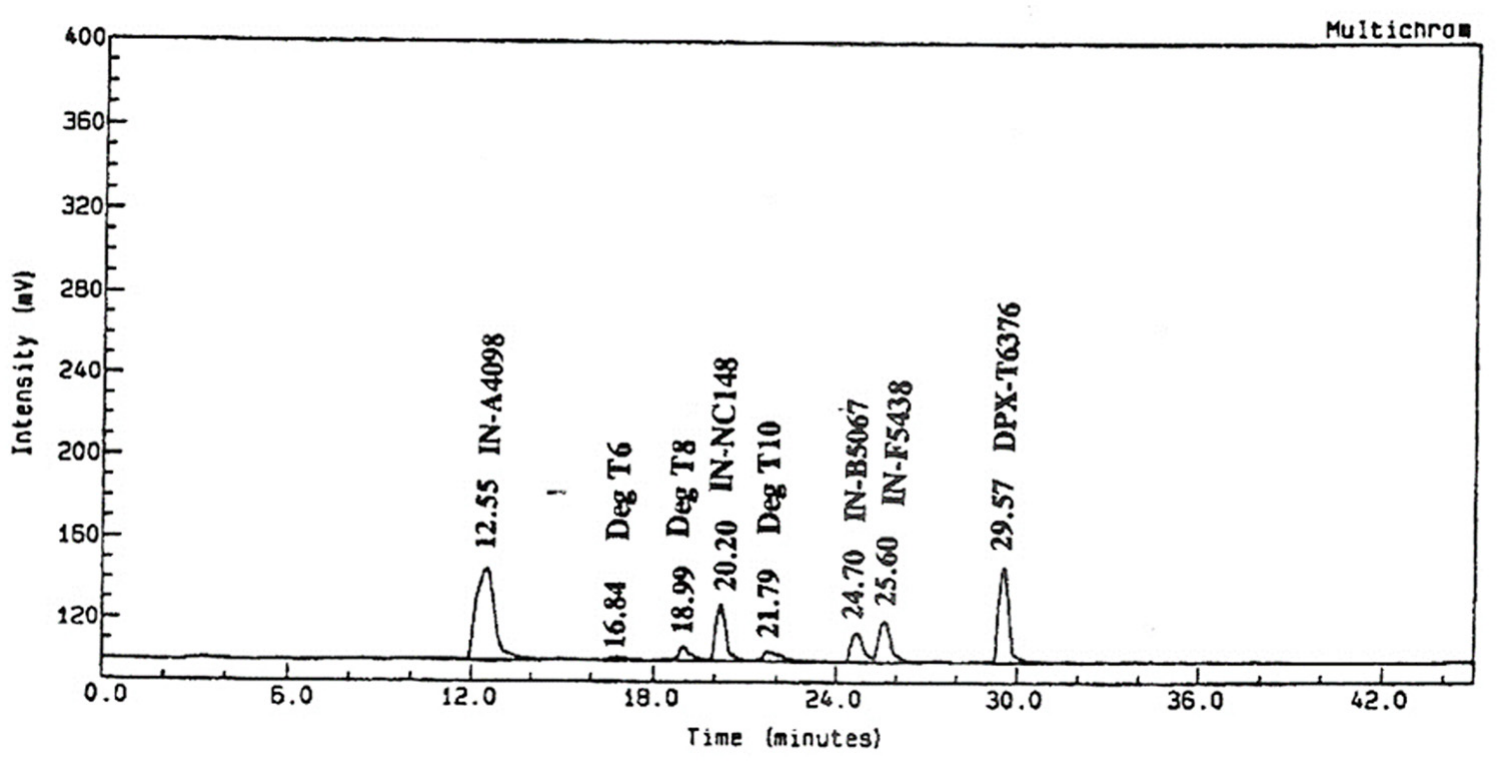
^14^C-radiochromatogram of ^14^C-triazine metsulfuron-methyl in the non-sterile system (in day 30).

In the non-sterile system, throughout the duration of the study seven metabolites were observed, of which one was < 5% (T5). The day -0 chromatogram from the fraction collection of the HPLC analysis is presented in Fig 7.

**Fig 8:**
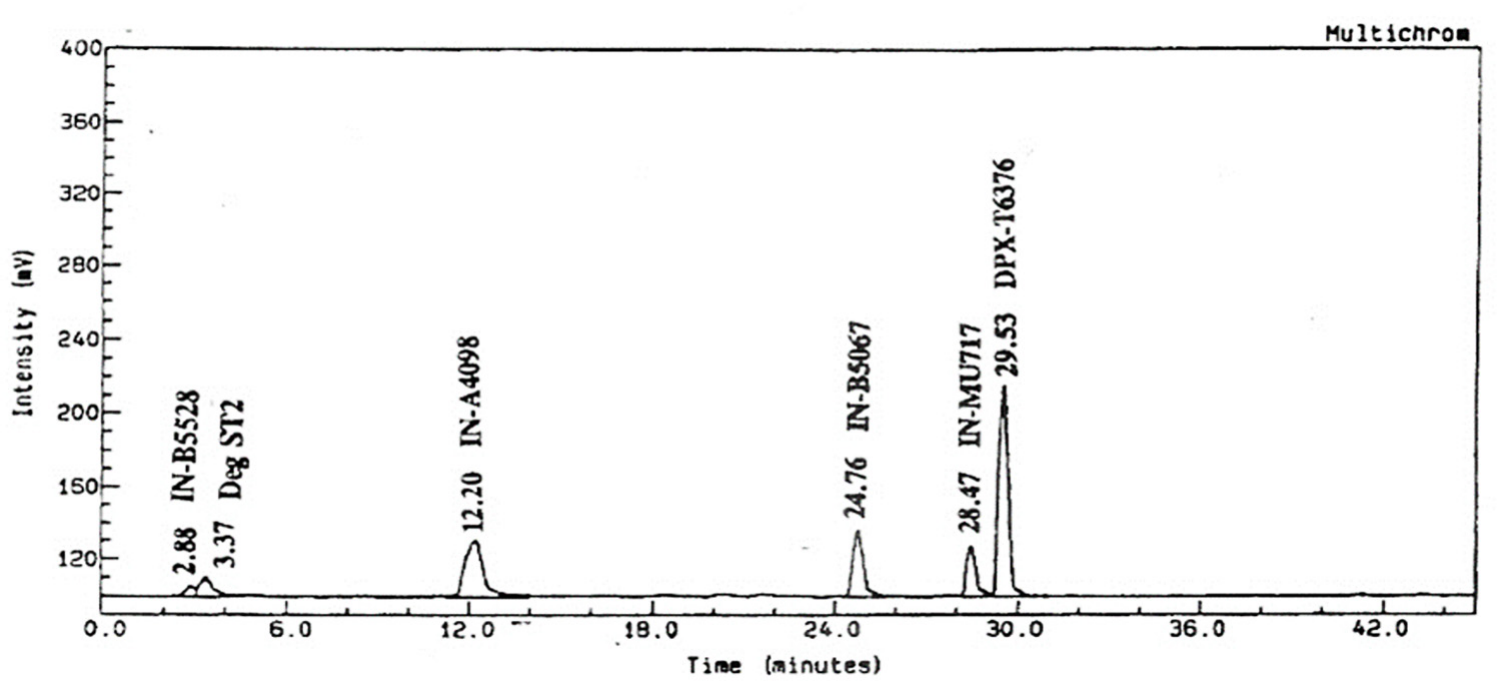
^14^C-radiochromatogram of ^14^C-triazine metsulfuron-methyl samples in the sterile test system (day 30).

The peak of the metabolites T1, T2, T4, T6 and T7 corresponded to IN-B5528, IN-A4098, IN-B5685, IN-NC148, IN-B5067 and IN-F5438 by relative retention time (RRI) comparison. T3 was not identified in the present study as it was < 10%. The metabolite IN-B5528 reached a maximum of 12.8% on day 10. At subsequent time points it was not detected. The metabolites IN-A4098 was not detected until day 14. It reached a maximum of 26.6% on day 30 and declined to 23.8% on day 60. T3 was consistently present from day 7 until day 60 within the range of 3.3% and 6.9%. No specific trend was observed for the metabolite IN-NC148, which was detected from day 14 onwards until day 60 within the range of 1.5% and 6.5%. The metabolite IN-B5067 reached a maximum of 16.5% on day 10 and declined to < 5% on day 60. The metabolite IN-F5438 reached a maximum of 5.3% on day 30 and declined to < 5% on day 60. In the sterile system, six metabolites were observed over the duration of the study, two of which were at levels < 5% (SP3 and SP4). The day 30 chromatogram from fraction collection on HPLC analysis is presented in Fig 8.

The peaks of the metabolites ST1, ST2, ST5 and ST6 corresponded to IN-B5528, IN-A4098, IN-B5067 and IN-MU717 by relative retention time (RRI) comparison. The metabolite IN-B5528 reached a maximum of 8.3% on day 7 and declined to < 5% on day 60. The metabolite IN-A4098 was not detected until day 14, after which it increased and reached a maximum of 24.3% on day 60. Metabolite IN-B5067 reached a maximum of 17.3% on day 21 but declined to 10.1% on day 60. Metabolite IN-MU717 reached a maximum of 15.2% on day 30 and declined to 11.3% on day 60. The metabolite IN-B5528, IN-A4098, IN-NC148 and IN-B5067 were observed in both the non-sterile and sterile systems.

#### Metabolic Pathway of Radiolabelled Metsulfuron-methyl in Aerobic Soil

**Fig 9:**
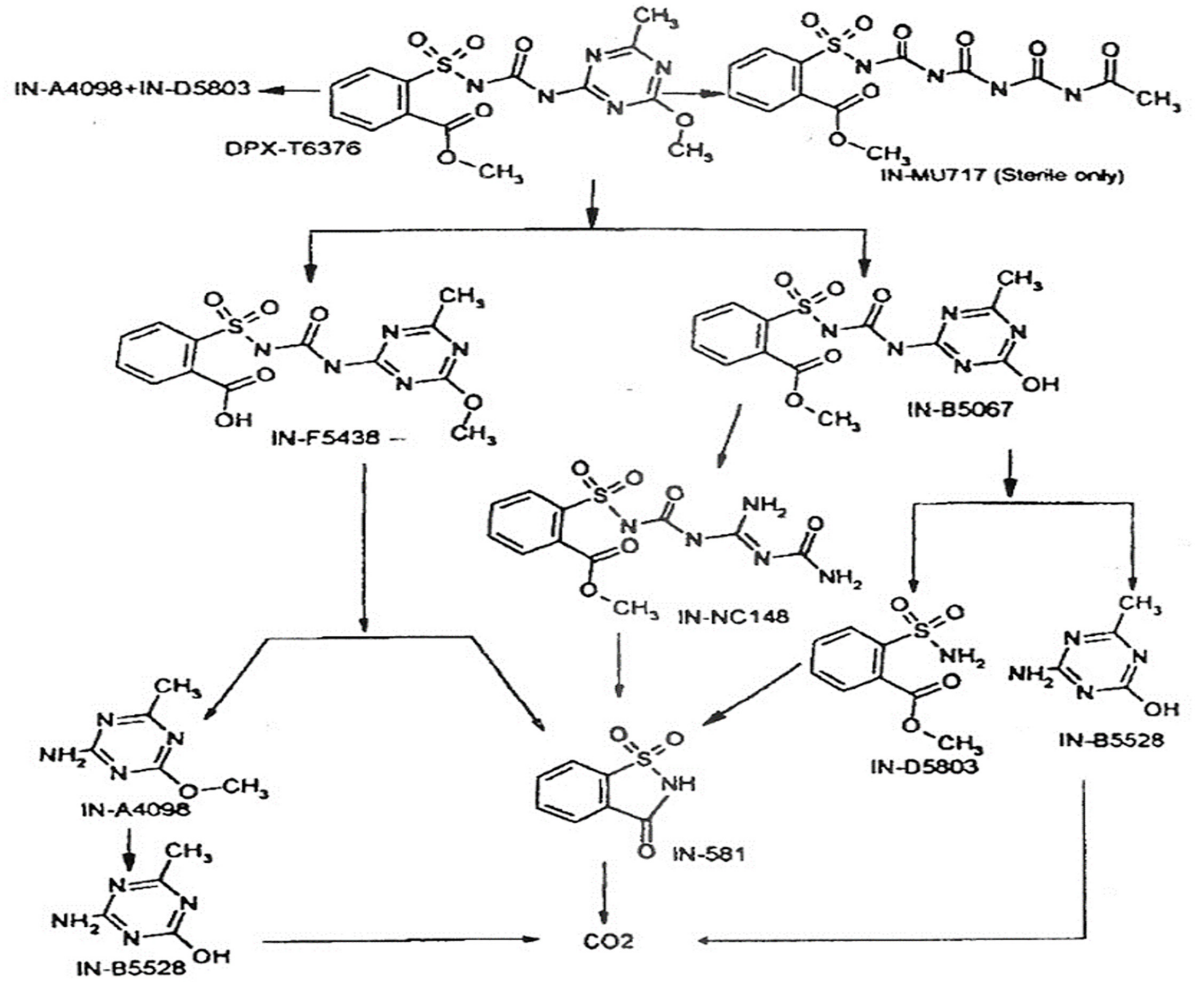
Proposed metabolic pathways of metsulfuron-methyl in Bernam Series.

The proposed degradation pathway of metsulfuron-methyl in soil under aerobic conditions is summarised in Fig 9. The major metabolites in the non-sterile system that include triazine moieties are IN-B5067, IN-F5438 and IN-NC148. Metabolite IN-B5528 was contained only in the triazine moiety. The ^14^C-triazine metsulfuron-methyl was mineralised to ^14^CO_2_ extensively in the non-sterile soils. In the sterile system, the major metabolites included both the phenyl and triazine moieties together and were IN-B5067 and IN-MU717. Metabolites IN-A4098 and IN-B5528 were contained only in the triazine moiety.

Cleavage of the sulfonylurea linkage, commonly found in other sulfonylurea herbicides [14, 24, 25], results in the formation of metabolites, IN-B5528 and IN-D5803. The urea bridge cleavage occurs rapidly in acidic aqueous solutions in the absence of micro-organisms, but is slow at neutral pH. In the non-sterile soil, this cleavage proceeds through both the abiotic bridge hydrolysis as well as the microbial mediated hydrolysis [26]. O- Demethylation of the triazine moiety of metsulfuron-methyl may be a chemical or microbial process (or both) since metabolite IN-B5067 was found in both the sterile and non-sterile soils. Metabolite IN-MU717 was unique to the sterile soil conditions suggesting that it is an abiotic hydrolysis product. The triazine “ring-opened” degradation products analogous to IN-MU717 have been reported for other sulfonylurea herbicides such as prosulfuron [27], thifensulfuron-methyl [28] and chlorsulfuron [29] in hydrolysis studies. A triazine “ring-opened” degradation product of metsulfuron-methyl was reported in an aqueous hydrolysis study at pH 5 and under sterile soil condition in a soil degradation study [4, 30]. Both hydrolysis and soil studies reported the same triazine “ring-opened” degradation products with an elemental composition of C13H14N4O8S which is equivalent to the metabolite IN-MU717, as noted in the present study. Metabolite IN-MU717 was not observed in the non-sterile soil samples. A study by Cessna [28] suggested two possible explanations: Firstly, the metabolite IN-B5067 may be further degraded preferentially into metabolite IN-NC148 by microbial transformation instead of being degraded to metabolite IN-MU717 by chemical hydrolysis. The microbial enzymatic reaction from metabolite IN-B5067 to IN-NC148 may be faster than the hydrolysis reaction to IN-MU717. Secondly, metabolite IN-MU717 may have been formed but got degraded microbially to undetectable levels by the time the samples were taken. Metabolite IN-NC148 appears to be a biotransformation product since it was not detected in the sterile soil. The same finding was reported by [31, 32]. An analogous metabolite was observed in a chlorsulfuron soil dissipation study [33, 34]. A possible mechanism for the formation of IN-NC148 can be proposed as follow: The first step appears to be O-demethylation of the triazine ring to form the metabolite IN-B5067, as this metabolite was observed initially, then it declined when the presence of IN-NC148 gradually increased (Fig 9). Enzyme-mediated hydrolytic bond cleavage of two of the triazine ring C-N bonds will then have to take place to arrive at the carbamoyl guanidine structure of IN-NC148.

### Conclusion

Metsulfuron-methyl degraded faster in acidic soil in the dark at 30°C under the non-sterile system, compared to degradation in the sterile system. The estimated DT_50_ and DT_90_ values of metsulfuron-methyl in the non-sterile system using the first-order model were approximately 13 days and 44 days, respectively. In the sterile system, the estimated DT_50_ and DT_90_ values of metsulfuron-methyl were approximately 31 days and 70 days, respectively. This result is consistent with previous reports. The fact that the DT_50_ values of metsulfuron-methyl in the non-sterile system were lower than the values in sterile system suggest that biological degradation (by soil micro-organisms) significantly contributed to the dissipation of the compound. The dissipation of metsulfuron-methyl in the soil involves both chemical and microbial processes, and this is true for other sulfonylurea herbicides as well. Degradation of metsulfuron-methyl in an acidic soil environment is relatively rapid because of the sulfonylurea bridge hydrolysis which diminishes in alkaline conditions. The principal degradation product after 60 days was carbon dioxide. The cumulative ^14^CO_2_ in ^14^C-phenyl and ^14^C-triazine labelled metsulfuron-methyl levels in the non-sterile system were approximately 28% and 24% of the applied radioactivity respectively. Lower cumulative levels of ^14^CO_2_ were recorded in the sterile system, which accounted for approximately 14% of the applied radioactivity and 0.5% of the applied radioactivity in the ^14^C-phenyl treatment. The different rates of mineralization indicate that the phenyl moiety is more susceptible to microbial attack than the triazine moiety on the molecule. The major routes of degradation are O-demethylation, sulfonylurea bridge cleavage and triazine “ring opened”. Two triazine “ring-opened” products, namely metabolite IN-MU717 in the sterile system, and metabolite IN-NC148 in the non-sterile system, were identified in the study. Acetyl triuret IN-MU717 and analogous compounds have been observed in the hydrolysis of metsulfuron-methyl and related sulfonylurea herbicides, as discussed previously, while the carbamoyl guanidine IN-NC148 has been reported in a study by Li *et al*. [33]. Microbial metabolism best explains the formation of IN-NC148 while chemical hydrolysis leads to the formation of IN-MU717. The potential for soil persistence of the sulfonylurea herbicides containing the triazine moiety breakdown products has been previously the subject of speculation; however, using sensitive LC/MS technology, the structures of the major triazine ring breakdown products have been definitively elucidated. The results indicate that the triazine ring opening did occur and that hydrolytic and microbial mechanisms were operable.
